# Parallel Changes in Structural and Functional Measures of Optic Nerve Myelination after Optic Neuritis

**DOI:** 10.1371/journal.pone.0121084

**Published:** 2015-05-28

**Authors:** Anneke van der Walt, Scott Kolbe, Peter Mitchell, Yejun Wang, Helmut Butzkueven, Gary Egan, Con Yiannikas, Stuart Graham, Trevor Kilpatrick, Alexander Klistorner

**Affiliations:** 1 Department of Ophthalmology, Save Sight Institute, University of Sydney, Sydney, Australia; 2 Centre for Neuroscience, University of Melbourne, Melbourne, Australia; 3 Department of Neurology, Royal Melbourne Hospital, Melbourne, Australia; 4 Department of Medicine, University of Melbourne, Melbourne, Australia; 5 Department of Neurology, Box Hill Hospital, Melbourne, Australia; 6 Monash University, Melbourne, Australia; 7 Department of Radiology, Royal Melbourne Hospital, Melbourne, Australia; 8 Department of Neurology, Concord Hospital, Sydney, Australia; 9 Australian School of Advanced Medicine, Macquarie University, Sydney, Australia; University of Houston, UNITED STATES

## Abstract

**Introduction:**

Visual evoked potential (VEP) latency prolongation and optic nerve lesion length after acute optic neuritis (ON) corresponds to the degree of demyelination, while subsequent recovery of latency may represent optic nerve remyelination. We aimed to investigate the relationship between multifocal VEP (mfVEP) latency and optic nerve lesion length after acute ON.

**Methods:**

Thirty acute ON patients were studied at 1,3,6 and 12 months using mfVEP and at 1 and 12 months with optic nerve MRI. LogMAR and low contrast visual acuity were documented. By one month, the mfVEP amplitude had recovered sufficiently for latency to be measured in 23 (76.7%) patients with seven patients having no recordable mfVEP in more than 66% of segments in at least one test. Only data from these 23 patients was analysed further.

**Results:**

Both latency and lesion length showed significant recovery during the follow-up period. Lesion length and mfVEP latency were highly correlated at 1 (*r* = 0.94, p = <0.0001) and 12 months (*r* = 0.75, p < 0.001). Both measures demonstrated a similar trend of recovery. Speed of latency recovery was faster in the early follow-up period while lesion length shortening remained relatively constant. At 1 month, latency delay was worse by 1.76ms for additional 1mm of lesion length while at 12 months, 1mm of lesion length accounted for 1.94ms of latency delay.

**Conclusion:**

A strong association between two putative measures of demyelination in early and chronic ON was found. Parallel recovery of both measures could reflect optic nerve remyelination.

## Introduction

Axonal degeneration is the major determinant of progressive neurological disability in MS [1]. Acute inflammatory demyelination is one of the principal causes of axonal transection and subsequent degeneration in MS lesions (see [2] for review). Chronic demyelination could also contribute to axonal loss by making axons more vulnerable to physiological stress [3], while the lack of trophic support from myelin and the disruption of normal axon-myelin interaction may further add to axonal degeneration [2]. Remyelination strategies could, therefore, be highly effective in preventing axonal loss [4] and potentially ameliorating MS disability. Spontaneous remyelination, while an early and frequent event in MS [5] is often incomplete and strategies that promote remyelination are needed. A number of approaches to promote myelin repair [6,7] have made significant progress in experimental models and *in vivo* measures that can assess the therapeutic and biological efficacy of putative remyelinating treatments are necessary in order for a transition to clinical therapy. As such, objective measures of demyelination and remyelination are critical in the assessment of such interventions.

The visual evoked potential (VEP) [8] can serve as an objective functional measurement of the visual pathway. While VEP amplitude reflects the number of functional afferent fibres reaching striate cortex (V1) and the degree of synaptic activity in V1, VEP latency delay qualitatively represents the effect of conduction change in the visual system [9]. Since speed of conduction slows down dramatically in demyelinated fibers due to change from saltatory to continuous mode, the initial latency prolongation after an episode of acute optic neuritis (ON) reflects the degree of demyelination (which our group has recently confirmed in an animal model[10]). On the other hand, the initial size of the optic nerve lesion, as measured by MRI, reflects the extent of inflammatory demyelination and, therefore, is likely to be proportional to the degree of myelin loss.

However, attempts to correlate those two potential measures of optic nerve demyelination have produced controversial results in the past [11–13]. Davies and co-workers[12], in a cross-sectional study of 25 MS patients (mostly without a history of ON), demonstrated significant correlation between optic nerve lesion length and latency delay of the full-field VEP using inter-eye asymmetry. However, Youl and colleagues [11] reported no correlation between lesion length and VEP latency at onset or 1 month after onset of acute ON. The authors attributed their results to the poor MRI resolution, the effect of oedema on the radiologically identified lesion, and too small a sample size. Trip and coworkers [13], in a study of 25 patients with a previous episode of ON, also did not find correlation between lesion length and VEP latency delay. There was, however, a considerable range of time since ON onset between subjects (1–9 years) and a selection bias towards patients with incomplete visual recovery, which is often associated with absent VEP responses. Other factors, which possibly contributed to poor correlation, include technical issues (such as low MRI resolution), small sample size and patient’s selection (bias toward severe disease, variable time since onset of ON).

The conventional full-field VEP, used in all previous studies may also potentially be a confounder as it provides a summed response of all neuronal elements stimulated, and, is greatly dominated by the macular region due to its cortical overrepresentation. In addition, the waveform of the full-field VEP is prone to unpredictable change depending on the part of the nerve affected. This can lead to detection of apparent rather than real latency delay and considerable waveform distortion. Use of the recently developed multifocal VEP (mfVEP) removes limitations of full-field stimulation by providing simultaneous, but independent stimulation of a plurality of visual field locations. It minimises the possibility of wave distortions from variably oriented parts of the visual cortex, providing a more accurate evaluation of conduction along the optic nerve [14].

In the current study we aimed to examine the relationship between structural and functional measures of optic nerve integrity, which are potentially related to demyelination, in a cohort of patients with acute ON using a combination of mfVEP and high resolution MRI. In addition, evolution of both measures was studied during 12 months after onset of ON to determine if the relationship was maintained.

## Methods

### Subjects

Thirty adults with a recently diagnosed, first episode of isolated unilateral ON were recruited from a tertiary ophthalmology hospital. All patients had a cerebral MRI scan with at least two hyperintense lesions consistent with demyelination, thus placing them at high risk of subsequent development of MS [15]. The unaffected eye was confirmed to be normal both clinically and using mfVEP. Patients were recruited within two weeks of symptom onset and were studied at 1, 3, 6 and 12 months after the onset of acute ON. Optic neuritis was diagnosed using standard clinical criteria [16].

This study was conducted in accordance with the Declaration of Helsinki and was approved by the human research ethics committee of the Royal Victorian Eye and Ear Hospital (Study number 07/740H). Study participants provided voluntary, written consent.

### Clinical assessments

Best corrected visual acuity (VA) was measured at 1 month using Sloan high contrast (100%) and low contrast letter acuity charts (LCLA) (2.5% and 1.25%) at 2m[17]. Snellen VA equivalents (documented in LogMAR notation) were determined from 100% contrast charts. For LCLA, the numbers of letters correctly identified (maximum 60/chart) were recorded for each eye. A detailed ophthalmological examination was performed at baseline.

### MR Imaging and Analysis

**Fig 1:**
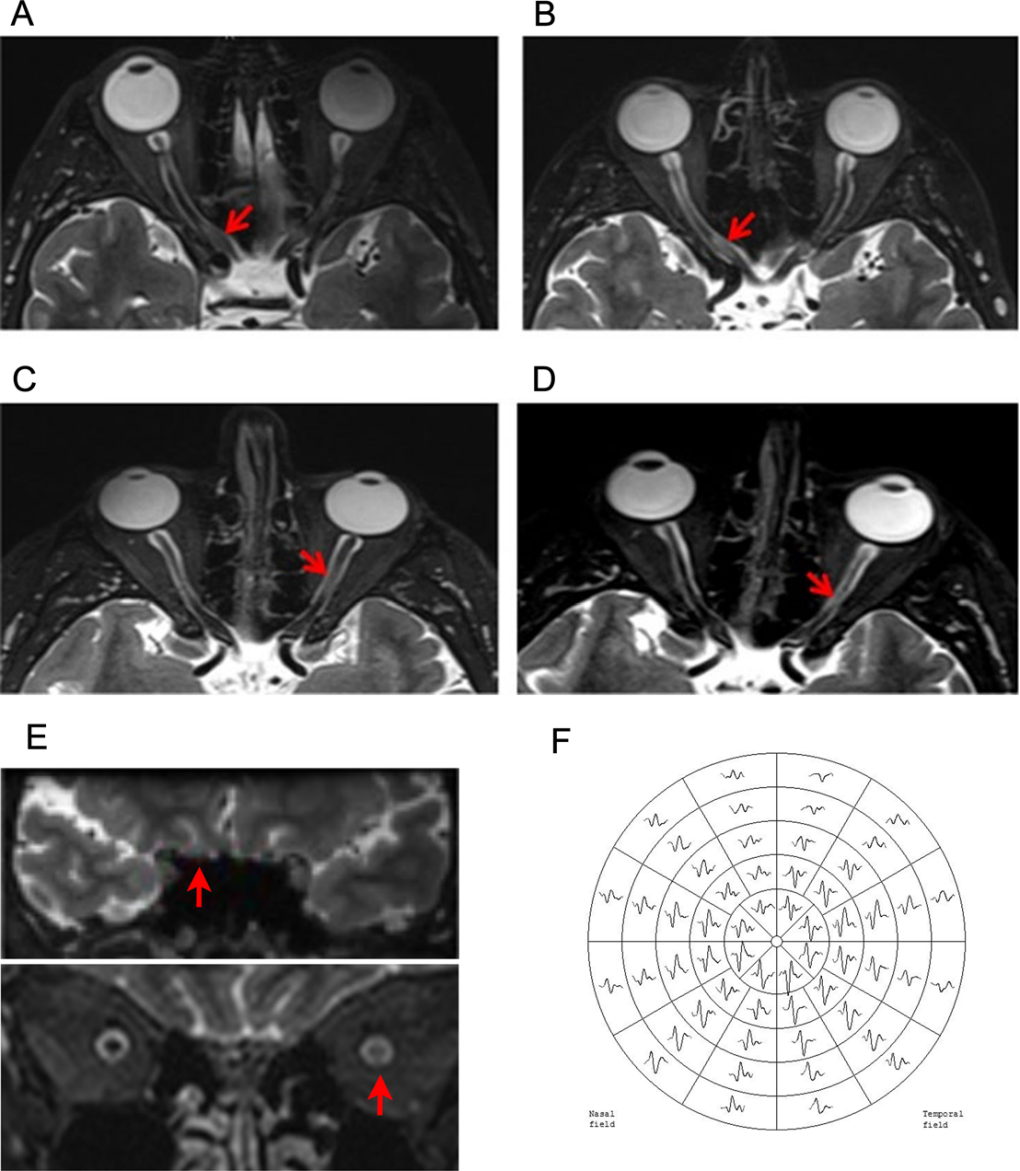
Example of optic nerve MRI lesions after ON and mfVEP recording. A-B: Intra-cannalicular lesion at 1 month (A) and 12 months (B) after ON. Lesion length decreased from 9.9 mm to 7 mm. C-D: Intra-orbital lesion at 1 month (C) and 12 months (D) after ON. Lesion length decreased from 11.3 mm to 5.5 mm. E: Coronal views of intra-cannalicular (above) and intra-orbital (below) lesions. F: Example of mfVEP trace.

Participants were imaged using a Siemens 3T MRI scanner with a 32-channel head-coil. To minimize eye movement during optic nerve scanning, subjects were instructed to perform a simple eye fixation task [18]. For lesion identification, each optic nerve was imaged using a 3D T2-weighted sequence (TR = 6000ms; TE = 403ms; TI = 2100ms; flip angle = 120°; ETL = 123; matrix size = 256 x 222; FOV = 256 x 222mm^2^; slice thickness = 1mm). Optic nerve lesions were identified by a neurologist on coronal T2-DICOM images using Osirix Open Source Software [19] and was verified by a blinded neuroradiologist (see examples in Fig 1A–1E). Lesion length was calculated as the square root of the sum of the square sagittal length and the square coronal length to correct for nerve tortuosity. Twenty randomly selected nerves were rated twice, 1 month apart to assess intra-observer reliability [20].

### mfVEP recording and analysis

Multifocal VEP testing was performed using the Accumap (ObjectiVision Pty. Ltd., software: Opera, Sydney, Australia) employing standard stimulus conditions [21] that provided recordings from 58 segments of the visual field (see example in Fig 1F). Monocular recordings were completed for 10 to 12 runs until a sufficient signal to noise ratio was reached. Four gold cup electrodes were placed around the inion and used for bipolar recording from four channels: superior and inferior, left and right, and obliquely between horizontal and inferior electrodes. Amplitude analysis: the wave of maximal amplitude among the four channels was automatically selected to create a combined topographic map. Latency analysis: 1) there were four traces for each eye recorded for every individual segment at each test 2) amplitude of the traces from all four channels of both eyes of all four tests from single segment of the visual field was analysed as described above (4 channels x 2 eyes x 4 tests = 32 traces) and the amplitude of the largest wave was recorded 3) the second peak of the largest wave was automatically determined for latency measurement 4) the same peak (minimum or maximum) were then used for latency analysis for that particular segment in both eyes in all four tests. Latency of a particular segment was included in averaged analysis only if amplitudes in both eyes of all four tests were recordable.

### Statistical Analyses

Mean values were calculated for all parameters for both patient nerves at each time point. Intra-observer reliabilities for optic nerve lesion length were tested using the Bland and Altman method [20]. Use of inter-eye asymmetry (calculated as the difference between the latencies of affected and fellow eyes in milliseconds), rather than absolute values, minimises the effects of between-subject variability, and is a better measure of disease-induced changes than absolute latency values, as long as the fellow eye is not affected [22]. It also eliminates the contribution of possible post-chiasmal damage [12].

Partial correlation corrected for age and sex was used to compare lesion length and mfVEP data. Statistical analyses were performed using IBM SPSS 20.

## Results

Demographic data are presented in Table 1. Ten clinically isolated syndrome patients had a second non-ON relapse during the follow up period. No patient was on disease modifying treatment for MS at baseline, but 46.7% (n = 14) were on treatment by 12 months.

**Table 1:** Demographic and visual acuity data at 1 month.

Age	35.3+/-9.6
Gender (F/M)	2.8/1
LogMAR VA	0.61 +/- 0.36
2.5% LCLA (letters correct out of 60)*	3.6 +/- 9.0
1.25% LCLA (letters correct out of 60)*	6.2 +/- 10.2

VA = visual acuity, LCLA = Low contrast letter acuity

### Multifocal VEP

By one month, the mfVEP amplitude had recovered sufficiently for latency to be measured in 23 (76.7%) patients. Seven patients had no recordable mfVEP in more than 66% of segments in at least one test, which prevented latency calculation. Therefore, only data from 23 patients was analysed further (Table 2). There was significant latency improvement during the follow-up period (average latency asymmetry shortening 8.2+/-5.6 ms, p<0.0001). Latency delay at 1 and 12 months correlated well (*r* = 0.94, p<0.0001). Recovery of mfVEP latency was fastest during the first 2 months of the follow-up period.

**Table 2:** Averaged mfVEP latency asymmetry and lesion length asymmetry values at all time points.

	1 month	3 months	6 months	12 months
Latency, ms	17.5+/-10.3	14.0+/-10.6	11.3+/-8.9	9.3+/-7.2
Lesion length, mm	9.9+/-5.5	8.7+/-5.2	7.0+/-4.7	4.5+/-4.3

### Optic nerve MRI

A single lesion was identified in 20 out of 23 (87%) affected optic nerves. Nine (45%) lesions were intra-orbital, 4 (20%) intra-cannalicular, 4 (20%) intra-orbital and intra-cannalicular and 3 (15%) were intra-cannalicular to prechiasmal. In the remaining three patients no discrete lesions were detected on the 1-month MRI. The mean intra-observer measurement difference was 0.60 mm (SD 1.4mm). 95% of measurements fell between the 95% limitations of agreement.

The T2 lesion length reduced significantly during the follow up period with the average lesion length shortening from 1 to 12 months being 4.4 +/-3.5 mm, p<0.0001.(see Table 2). There was a strong correlation between initial and final lesion length (*r* = 0.76, p<0.001). Speed of T2 lesion shortening was similar during entire follow-up period.

### Relationship between lesion length and latency delay

**Fig 2:**
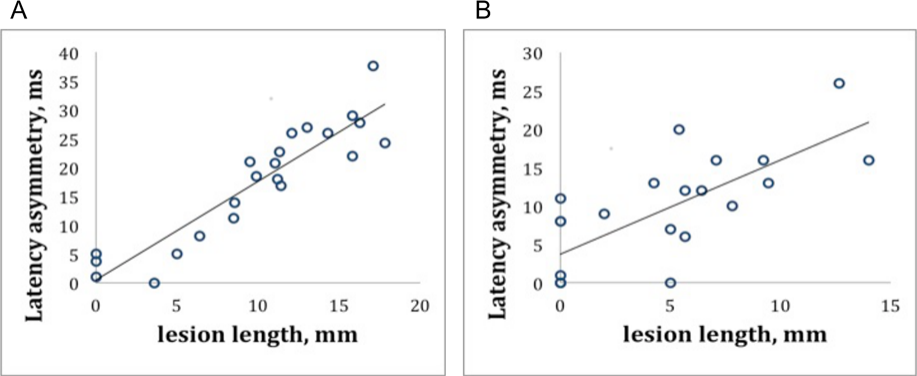
Relationships between mfVEP latency asymmetry and optic nerve lesion length at 1 (A) and 12 months (B). A. Multifocal VEP latency asymmetry (in milliseconds (ms) and lesion length (in millimeters (mm) at 1 month after acute ON were highly correlated. B. Decrease in mfVEP latency asymmetry (ms) and shortening of lesion length (mm) remained signficantly correlated 12 months after ON.

**Fig 3:**
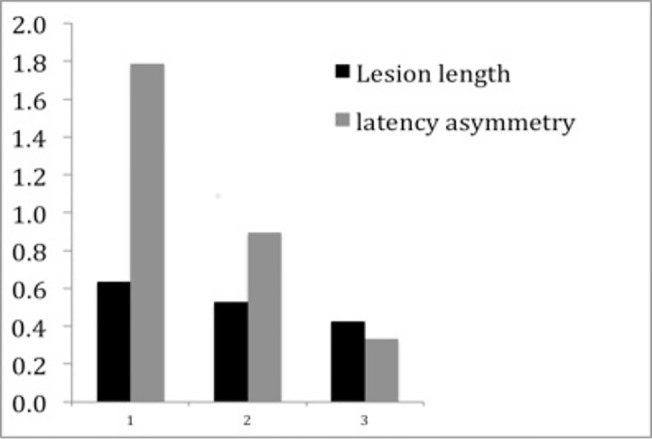
Monthly rate of latency asymmetry recovery and lesion shortening. The monthly recovery for each interval was calculated, indicating that early on, speed of recovery is much faster for latency but less so for lesion size. y axis: change in mfVEP latency asymmetry (ms) or lesion length (mm) between time points. x-axis: Follow-up periods: 1 = 1–3 months, 2 = 3–6 months and 3 = 6–12 months.

There was a highly significant correlation between lesion length asymmetry and mfVEP latency asymmetry at 1 month (*r* = 0.94, p = <0.0001, adjusted for age and sex) (Fig 2A) that remained significant at 12 months (correlation at 12 months: *r* = 0.75, p < 0.001) (Fig 2B). Moreover, reduction of lesion length moderately, but significantly, correlated with shortening of the mfVEP latency (*r* = 0.48, p = 0.03, age and sex adjusted). While speed of latency recovery (calculated as change in latency asymmetry between follow-up time points) was visibly faster during early follow–up period, rate of lesion length reduction (calculated as change in lesion length between follow-up time-points) remained relatively constant throughout entire monitoring (Fig 3). At 1 month, the latency delay was calculated to be worse by 1.76 ms for every additional 1 mm of the lesion length. This relationship slightly changed at 12 months where 1 mm of the lesion length was associated with 1.94 ms of the latency delay.

We calculated sample sizes (based on 80% power, p<0.05) to estimate the utility of using latency recovery and lesion length shortening as potential measures of remyelination in a clinical trial setting. To detect a 50% treatment effect based on latency shortening between 1 and 12 months would require a sample of 15 patients while 43 patients would be needed to show a 30% treatment effect using latency recovery. Demonstrating a 50% treatment effect using shortening of T2 lesion length from 1 to 12 months would require a sample of 20 patients whereas demonstrating a 30% treatment effect would require 57 patients over the same time period. Any possible residual oedema would be resolved at the 3-month time point and demonstrating a 50% treatment effect between 3 and 12 months would require 31 patients on mfVEP latency shortening 26 patients using lesion shortening. However, to detect a 30% treatment effect from 3 to 12 months would require larger cohorts of 82 patients for latency shortening and 68 patients for lesion length shortening.

## Discussion

This study examined the correlation between optic nerve lesion length and mfVEP latency delay in a sizable cohort of ON patients at high risk of developing MS over a 12-month period. The use of the mfVEP, as well as the identification of optic nerve lesions using high-resolution 3T MRI enabled us to examine the relationship between lesion length and latency delay with high degree of precision. We found a strong association between these two measures in early post-ON period. Furthermore, we demonstrated parallel recovery of both measures over the year after onset of ON.

It is understood that the visibility of MS lesions on T2-weighted images is the result of increased water content and tissue restructuring. The histopathological specificity of altered MRI signals, however, is poor. It has been suggested that a combination of several factors contribute to T2 hyperintensity at various stages of lesion evolution [23]. During the acute stage, the MS lesion is characterised by almost total demyelination, extensive inflammatory reaction, axonal transection, parenchymal edema and expansion of the extracellular space secondary to myelin loss [24]. The area of inflammatory demyelination is typically well demarcated and, despite some peri-plaque oedema, is largely proportional to the size of T2 hyperintensity [25].

Acute lesions gradually evolve towards the resolution of inflammation and development of gliosis. Cytoplasm-rich glial cells [26] fill-up the chronically demyelinated region, maintaining increased water content in plaque tissue and T2 hyperintensity [27]. Therefore, even though chronic demyelination *per se* may have little effect on the intensity of the T2 image, it is indirectly involved in the formation and spatial extent of the chronic MRI-defined lesion. Tissue restructuring caused by axonal loss, on the other hand, extends far beyond the lesion in a form of Wallerian and retrograde axonal degeneration and, therefore, is unlikely to have a significant bearing on MRI-defined lesion border.

Conduction, which is initially blocked in the surviving, but acutely demyelinated axons, typically recovers within 3–4 weeks due to redistribution of ion channels, which coincides with functional improvement. However, change from saltatory to continuous mode of signal transmission results in significant reduction of the conduction velocity within the demyelinated area, while outside the lesion the normal conduction speed is largely maintained [28]. As a result, the latency of the VEP is delayed proportionally to the extent of the demyelinated area. This direct association between latency delay and degree of optic nerve myelination has recently been confirmed using an animal model of optic nerve demyelination [10,29].

Both lesion length and mfVEP latency delay relate to the amount of myelin loss and it is therefore not surprising that both measures correlate well. The correlation is particularly significant early after ON, which probably reflects the fact that, while structural measurement of demyelination becomes more indirect as time passes, conduction along optic nerve may also become affected by other factors, such as cortical plasticity. A study of inter-nuclear ophthalmoplegia in MS recently described a similar relationship between MRI lesion length in the medial longitudinal fasciculus and latency of the electrically evoked vestibulo-ocular reflex [30].

Structural and functional measures examined in the current study revealed a significant degree of improvement during the follow-up period. It has previously been suggested that MRI lesion shrinkage [31] and latency shortening [32] may be partially attributed to remyelination. The current study for a first time demonstrated the parallel nature of those changes suggesting that MRI-defined lesion reduction and mfVEP latency shortening may both reflect, at least to some degree, the process of remyelination.

Since first described in MS in 1965 [33], spontaneous remyelination is now believed to be an early and frequent phenomenon occurring in optic nerve, spinal cord and brain, even in presence of concurrent inflammation [5,34,35]. It varies widely from a rim of thin myelin sheaths around the edges of a lesion [36] to completely remyelinated lesions [37]. Remyelination in MS lesions is implemented by new oligodendrocytes that arise from oligodendrocyte precursor cells (OPC) (see [38] for review). The adult CNS contains a large number of OPC that retain significant proliferative capacity[39] and are scattered throughout white matter [40], including the optic nerve[41]. Under normal conditions OPCs remain relatively quiescent, but become activated in response to demyelination, start to proliferate and then migrate into the area of demyelination, where they finally differentiate and induce remyelination [38]. Since OPCs migrate into lesion from surrounding white matter, remyelination starts from (and often limited to) an external rim of the plaque [42]. Partially restored myelin sheaths refill extracellular space and replace some of the recently proliferated glial cells reducing water content in newly remyelinated areas and, therefore, decreasing hyperintensity of the T2 image. This process is probably responsible for MRI-defined lesion shrinkage.

Remyelination also reinstates saltatory conduction, which results in increased conduction speed. Remyelinated axons typically have a thinner and shorter myelin sheath than would be expected for a given diameter of axon [43]. However, while reduced myelin thickness and internode distance might decrease the robustness of saltatory conduction, it does not affect conduction velocity, which recovers fully within the remyelinated section of the axon [44]. This supports the hypothesis that mfVEP shortening is related to the restoration of myelin after an episode of acute ON and is also in line with previously described pattern of mfVEP latency recovery, which is consistent with the speed of OPC propagation through the section of optic nerve demyelination [45].

Since correlation between lesion shortening and latency recovery was only moderate, it suggests that other factors (such as cortical plasticity or axonal loss) may contribute to both the latency recovery of the mfVEP and the water content in the chronic lesion. There is an altered structure to newly formed myelin, which is thinner (and therefore, does not fill-up the entire space initially freed by demyelination), but it is fully functional and therefore completely restores the speed of conduction. This may also contribute to some structure-function discrepancy. This is supported by a weaker correlation between lesion length and latency delay at 12 months as compared to 1 month and a faster rate of latency recovery during the first 3 months.

Further studies using a larger patient cohort and expanded MRI methodology are needed to confirm these results. In particular, the inclusion of gadolinium-enhanced imaging could help to clarify the relationship between residual inflammation and demyelination and the effect of inflammation on the accuracy of mfVEP measurement and lesion characteristics. The duration of inflammatory oedema is approximately 1 month as indicated by the duration of gadolinium enhancement of brain lesions from MRI studies[46]. However, OCT studies indicate that oedema in the retinal nerve fibre layer usually persist for up to 3 months with slight residual oedema still present in some cases at 6 months after ON[47]. Advanced MRI techniques such as optic nerve diffusion tensor imaging [48] and magnetization transfer imaging [49] could also further enhance understanding of the pathological processes involved early after acute ON.
